# Biological Age Acceleration Associated with the Progression Trajectory of Cardio-Renal–Metabolic Multimorbidity: A Prospective Cohort Study

**DOI:** 10.3390/nu17111783

**Published:** 2025-05-24

**Authors:** Yixing Tian, Jinqi Wang, Tianyu Zhu, Xia Li, Haiping Zhang, Xiaoyu Zhao, Xinghua Yang, Yanxia Luo, Lixin Tao, Zhiyuan Wu, Xiuhua Guo

**Affiliations:** 1 Department of Epidemiology and Health Statistics, School of Public Health, Capital Medical University, Beijing 100069, China; tianyixing@mail.ccmu.edu.cn (Y.T.); wangjinqi@ccmu.edu.cn (J.W.); zhutianyu@mail.ccmu.edu.cn (T.Z.); yeszhang09@163.com (H.Z.); zhaoxiaoyu@mail.ccmu.edu.cn (X.Z.); xinghuayang@ccmu.edu.cn (X.Y.); lyx100@ccmu.edu.cn (Y.L.); taolixin@ccmu.edu.cn (L.T.); 2 Department of Mathematics and Statistics, La Trobe University, Melbourne 3086, Australia; x.li2@latrobe.edu.au; 3 Department of Nutrition, Harvard T.H. Chan School of Public Health, 655 Huntington Ave, Boston, MA 02115, USA; zhiyuanwu@hsph.harvard.edu; 4 National Institute for Data Science in Health and Medicine, Capital Medical University, Beijing 100069, China; 5 School of Medical and Health Sciences, Edith Cowan University, Perth 6027, Australia; 6 Beijing Key Laboratory of Environment and Aging, Capital Medical University, Beijing 100069, China

**Keywords:** biological age, diabetes, cardiovascular diseases, chronic kidney disease, multimorbidity

## Abstract

Objectives: Previous studies have confirmed that biological age (BA) acceleration is associated with single cardio-renal–metabolic diseases (CRMDs), typically including type 2 diabetes mellitus, cardiovascular disease, and chronic kidney disease. However, its association with progression to cardio-renal–metabolic multimorbidity (CRMM, coexistence of ≥2 CRMDs) and subsequent mortality remains unexplored. Methods: Using the multi-state model, we analyzed 278,927 UK Biobank participants free of CRMDs at baseline to investigate the association between BA acceleration—measured by phenotypic age (PhenoAge) and Klemera–Doubal method age (KDMAge)—and CRMM progression trajectory, from health to the first CRMD and then to CRMM and death. BA acceleration was the residual from regressing BA on chronological age; positive values indicated a biologically older individual. Results: PhenoAge acceleration showed stronger associations than KDMAge acceleration. Per the 1-SD increase in PhenoAge acceleration; HRs (95% CIs) were observed at 1.18 (1.17–1.19) for baseline to first CRMD; 1.24 (1.22–1.26) for first CRMD to CRMM; 1.25 (1.22–1.27) for baseline to death; 1.13 (1.11–1.15) for first CRMD to death; and 1.09 (1.06–1.12) for CRMM to death. Biologically older individuals by PhenoAge acceleration showed greater reductions in CRMD-free and total life expectancy than those by KDMAge acceleration. Age, socioeconomic status, education, smoking status, alcohol consumption, physical activity, and diet-modified risks for specific transitions. Conclusions: BA acceleration, particularly PhenoAge acceleration, relates to higher CRMM progression risk and shorter life expectancy. Combining BA acceleration with sociodemographic or lifestyle factors improves risk identification for specific transitions. BA acceleration offers the potential to guide CRMM prevention across its entire progression.

## 1. Introduction

As the global population rapidly ages, cardio-renal–metabolic multimorbidity (CRMM) has become a growing public health threat. CRMM refers to the coexistence of at least two cardio-renal–metabolic diseases (CRMDs), typically including type 2 diabetes mellitus (T2DM), cardiovascular disease (CVD), and chronic kidney disease (CKD) [1,2,3,4]. First, cardio-renal–metabolic disease (FCRMD) refers to the initial diagnosis of CVD, T2DM, or CKD as a single CRMD [2,5]. With longer life expectancy, patients with an FCRMD are more likely to develop additional CRMDs and thus progress to CRMM over their lifetime [3,4]. Compared to those with a single CRMD, patients with CRMM may experience greater disability, more complications, and a higher mortality risk [3,6,7,8]. Thus, identifying early risk factors for CRMM progression is a public health priority. Although chronological age (CA) is a common, unmodifiable risk factor for each component of CRMM (i.e., T2DM, CVD, and CKD), aging rates differ notably among individuals of the same CA [9]. To account for this difference, the concept of biological age (BA) has been proposed [10]. Compared to CA, BA can detect physiological changes earlier and predict age-related diseases and mortality risk more accurately [9].

Recent studies advocate that ideal BA algorithms should integrate aging information from multiple biological systems rather than rely on a single biomarker [11,12]. Phenotypic age (PhenoAge) [13] and Klemera–Doubal method age (KDMAge) [14] are representative examples of integrative BA algorithms that combine clinical biomarkers from multiple systems. These two algorithms are widely used for their superior accuracy in predicting morbidity and mortality and for their lower cost [13,15]. Mechanistic evidence suggests that biological aging may drive lipid metabolism dysregulation [16,17], inflammation [18], oxidative stress [19], and insulin resistance [20], possibly contributing to the shared progression of T2DM, CVD, and CKD. Thus, we infer that BA acceleration may be associated with every stage of CRMM progression. However, while previous studies have explored the association between BA acceleration and single CRMDs [21,22,23,24,25], they have not examined its association with the progression from CRMD to CRMM and subsequent mortality or associated life expectancy. Compared to mortality risk, life expectancy stands out as an intuitive public health indicator, facilitating public understanding and policy development. Studying these associations can effectively guide interventions, preventing CRMM progression and extending healthy lifespans.

To address this research gap, we developed two BA algorithms (PhenoAge and KDMAge) using UK Biobank data to (1) assess the association between BA acceleration and the progression from health to FCRMD and then to CRMM and death; (2) compare these associations in the transitions of different FCRMD types; (3) evaluate the association between these two BA measures and CRMD-free life expectancy with total life expectancy; and (4) investigate how sociodemographic and lifestyle factors modify the association between BA acceleration and CRMM progression to identify vulnerable populations.

## 2. Methods

### 2.1. Study Population

We used data from the UK Biobank, a large-scale cohort study that enrolled more than 500,000 UK residents aged 37 to 73 years. Detailed descriptions of UK Biobank measurement methods and data collection are available in previous studies [26]. Briefly, between 2006 and 2010, approximately 9.2 million individuals near 22 health assessment centers in England, Scotland, and Wales were invited to join the cohort. About 5.5% agreed to participate and provided written consent before enrollment. At baseline and during follow-up, participants provided data on lifestyle factors, health information, and physical measurements. Participants were excluded if they withdrew consent, had T2DM, CVD, or CKD before baseline, or lacked BA information at baseline. In total, 278,927 participants were included in the main analysis (Figure S1). The study was approved by the North West Multicenter Research Ethics Committee (reference number 11/NW/0382). This research was conducted using UK Biobank data under application number 88589.

### 2.2. Follow-Up for Cardio-Renal–Metabolic Diseases and Death

Incident cases of CRMDs (i.e., T2DM, CVD, and CKD) and death were ascertained using self-reports, primary care records, hospital admission records, and death registry records [1,2,27]. FCRMD was defined as the first occurrence of any CRMD [2,5], with diagnosis time based on the earliest recorded diagnosis among these conditions. CRMM was defined as the coexistence of at least two CRMDs [2,3,28], with diagnosis time based on the recorded diagnosis of a second CRMD during follow-up. Further details on CRMD diagnoses are provided in Table S1.

### 2.3. Assessment of Biological Age

We assessed BA using widely validated algorithms: PhenoAge [13] and KDMAge [14]. With reference to similar UKB studies [25,29], we identified 14 clinical biomarkers to calculate BA, covering multiple systems like immunity, inflammation, organ homeostasis, and metabolism. PhenoAge focused on immunity and inflammation, using nine biochemical parameters: lymphocytes, mean cell volume, serum glucose, red cell distribution width, white blood cell count, albumin, creatinine, *C*-reactive protein, and alkaline phosphatase [25,29]. KDMAge emphasized pulmonary function and cardiovascular metabolism, using forced expiratory volume in one second (FEV1), systolic blood pressure (SBP), and seven biochemical parameters: albumin, creatinine, *C*-reactive protein, alkaline phosphatase, total cholesterol, glycated hemoglobin, and blood urea nitrogen [25,29]. We excluded participants with missing data for any BA component (Table S2).

We trained the algorithm parameters in a reference sample and applied them to the UK Biobank data. The reference sample consisted of individuals aged 30 to 75 years from the National Health and Nutrition Examination Survey (NHANES) III cohort. PhenoAge was developed based on factors related to mortality risk. PhenoAge represents the predicted BA matching the average mortality risk in the reference sample [13]. KDMAge was derived from regression analyses of clinical biomarkers and CA, reflecting a predicted BA corresponding to normal physiological function in the reference sample [14]. BA acceleration was defined as the residual from a linear regression of BA on CA, reflecting the rate of biological aging [25,29]. We analyzed BA acceleration as a continuous variable and further dichotomized it, defining “biologically older” as KDMAge acceleration or a PhenoAge acceleration >0 (indicating faster aging) and “biologically younger” as ≤0. Table 1 summarizes the baseline characteristics of BA, its components, and BA acceleration. The algorithms for BA and the corresponding R code are available in the “BioAge” R package [30].

### 2.4. Covariates

Based on prior knowledge [2,5], the baseline covariates included age (years), sex (male/female), ethnicity (White/non-White), the Townsend deprivation index (≤median/>median), education level (low/high), body mass index (BMI, kg/m^2^), smoking status (never/former/current), alcohol consumption (none/moderate/heavy), physical activity (low/moderate/high), and dietary behaviors (unhealthy/healthy). In this study, a high educational level refers to a college or university degree or professional qualifications like nursing or teaching. Moderate alcohol consumption is defined as a daily intake of ≤14 g for females and ≤28 g for males. Physical activity is assessed using the International Physical Activity Questionnaire (IPAQ). Using the duration of activities (walking/moderate/vigorous), physical activity was converted into metabolic equivalents (MET) and categorized as low (<600 MET-minutes/week), moderate (600–3000 MET-minutes/week), or high (>3000 MET-minutes/week). Dietary behaviors were assessed according to recent cardiovascular health dietary recommendations [31]. Healthy dietary behaviors were defined as meeting at least five items (Table S3).

### 2.5. Statistical Analysis

Participant characteristics, grouped by incident disease states (CRMD-free, FCRMD, and CRMM), were summarized with mean and standard deviations (SDs) for continuous variables and the frequency and percentage for categorical variables. For covariates with missing data (0.12–21.69%, Table S4), multiple imputations by chained equations were applied to reduce inferential bias. Follow-up for each participant began at recruitment and continued until the earliest occurrence out of CRMD onset, death, loss to follow-up, or 19 December 2022.

**Figure 1:**
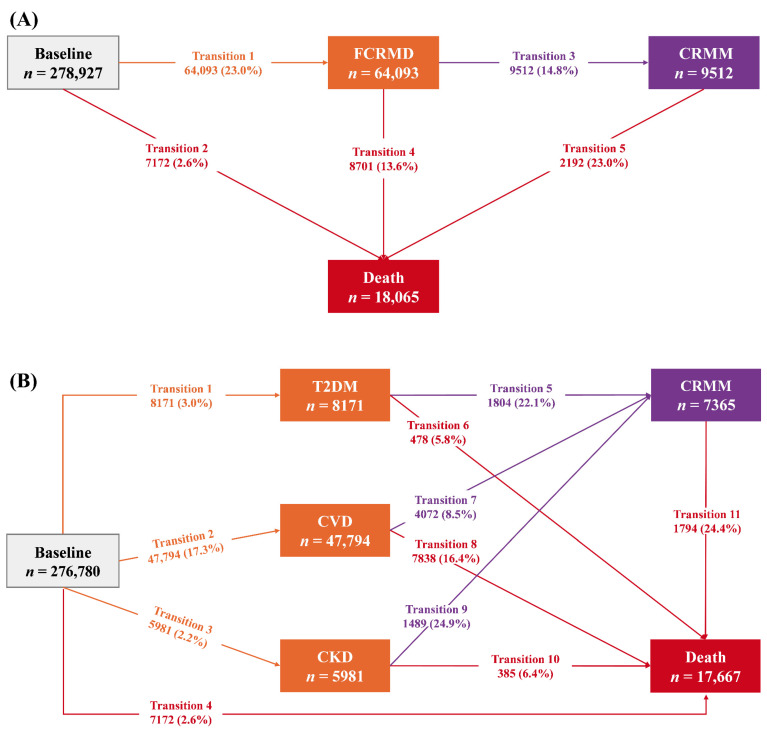
The number (percentage) of participants in different transitions. (**A**) The number (percentage) of participants in transition pattern A from baseline to FCRMD and then to CRMM and death; (**B**) the number (percentage) of participants in transition pattern B from baseline to one of T2DM, CVD, or CKD, and then to CRMM and death. Abbreviations: FCRMD: first cardio-renal–metabolic disease; CRMM: cardio-renal–metabolic multimorbidity (the coexistence of two or three CRMDs); T2DM: type 2 diabetes mellitus; CVD: cardiovascular disease; CKD: chronic kidney disease.

First, we used the Cox regression model to evaluate associations between BA acceleration and FCRMD, CRMM, and death. Second, to explore the association between the two BA accelerations and progression from baseline health to FCRMD, and then to CRMM and death, we applied the multi-state model. This model, an extension of the Cox regression model, estimates associations between risk factors and multiple disease states while accounting for competing risks [32,33]. Schoenfeld residuals verified the proportional hazards assumption. No violations were detected. Following previous research [2], five transition phases (transition pattern A, Figure 1A) were established: (1) baseline to FCRMD; (2) baseline to no *N*-CRMD death; (3) FCRMD to CRMM; (4) FCRMD to all-cause death; and (5) CRMM to all-cause death. If participants entered different states on the same date, we adjusted the prior state’s entry date to 0.5 days before the later state’s entry date, referencing prior studies [2,29,34,35]. Additionally, analyses specific to the various types of FCRMD were performed. In this analysis, eleven transitions (transition pattern B, Figure 1B) were established based on the three FCRMD types (T2DM, CVD, and CKD). In transition pattern B, participants with at least two CRMD diagnoses on the same date (*n* = 2147) were excluded as the sequence of CRMD events could not be determined. In total, 276,780 participants were included. To explore the dose–response relationship, we used a restricted cubic spline (RCS) analysis in transition pattern A, with knots at the 10th, 50th, and 90th percentiles.

Subsequently, using the “elect” R package and referring to previous studies [36,37,38], we calculated differences in CRMD-free and total life expectancy, using age (in years) as the time scale. Given notable sex differences in lifespan, we estimated life expectancy separately for males and females. To maintain estimate reliability and avoid unrealistic extrapolation [37], life expectancy predictions were restricted to ages 40, 50, and 60, covering the majority of the UK Biobank sample (99.62% aged <70 years) and representing middle age, late–middle age, and early old age.

To identify subgroups sensitive to BA acceleration, we performed subgroup analysis in transition pattern A based on sociodemographic characteristics (age, sex, Townsend deprivation index, and education) and lifestyle factors (BMI, smoking status, alcohol consumption, physical activity, and diet). Multiplicative interactions were tested using the likelihood ratio test.

To evaluate the stability of the results, we conducted several sensitivity analyses for transition pattern A: (1) for participants entering different states on the same date, the entry date for the prior state was calculated using four additional time intervals (0.5, 1, 3, and 5 years); (2) a transition directly from baseline to CRMM was added; (3) only White participants were included; (4) participants diagnosed with any CRMD within two years after enrollment were excluded; (5) further adjusting for the use of cholesterol-lowering and antihypertensive medications was conducted to assess their association with progression trajectory; (6) blood glucose was excluded from PhenoAge and glycated hemoglobin and creatinine from KDMAge to remove biomarkers directly tied to T2DM and CKD diagnoses; (7) participants with any of the following at baseline were excluded to mitigate the confounding effect of baseline glucose levels and kidney function: glucose ≥7.0 mmol/L with a fasting time ≥8 h, glucose ≥11.1 mmol/L with a fasting time <8 h, glycated hemoglobin ≥48 mmol/mol, an estimated glomerular filtration rate (eGFR) [39] <60 mL/min/1.73m^2^; or albuminuria ≥3 mg/mmol; (8) the association of BA acceleration per interquartile range (IQR) and increase in progression trajectory were evaluated; and (9) in the multi-state model, three states—specific FCRMD, specific two CRMM (coexistence of two CRMDs), and three CRMM (coexistence of three CRMDs)—were considered to investigate their associations with disease progression. This transition pattern is presented in Figure S2 (transition pattern C). All analyses were performed using R version 4.4.1.

## 3. Results

### 3.1. Descriptive Analysis

Table 1 outlines the characteristics of the total participants, CRMD-free participants, FCRMD patients, and CRMM patients. Patients with one or more CRMDs tended to be older, more often male and had a higher Townsend deprivation index, lower educational level, higher BMI, higher smoking rate, lower rate of moderate alcohol consumption, an older BA, and higher BA acceleration.

During a median follow-up of 13.79 years (IQR: 13.10–14.44), 64,093 participants (23.0%) experienced at least one CRMD. Among them, 9512 (14.8%) progressed to CRMM. During follow-up, 18,065 participants died: 8701 after FCRMD and 2192 after CRMM (Figure 1A). In the specific FCRMD analysis (excluding 2147 participants), 8171 (3.0%) patients developed incident T2DM; 47,794 (17.3%) developed incident CVD; and 5981 (2.2%) developed incident CKD (Figure 1B).

The mean (SD) values of PhenoAge acceleration and KDMAge acceleration were 6.51 (4.26) and −3.31 (9.76) years, respectively. A weak positive correlation was found between PhenoAge acceleration and KDMAge acceleration (with a Pearson correlation coefficient = 0.22; *p*  <  0.001).

### 3.2. BA Acceleration, CRMM and Mortality

PhenoAge acceleration was associated with the risks of FCRMD and CRMM using the Cox regression model (Table S5). After full adjustment, individuals classified as biologically older by PhenoAge acceleration showed a 64% increased risk of FCRMD (HR [95% CI]: 1.64 [1.59, 1.68]), a 137% increased risk of CRMM (HR [95% CI]: 2.37 [2.24, 2.50]), and a 107% increased risk of all-cause death (HR [95% CI]: 2.07 [1.99, 2.17]) compared with those classified as biologically younger. For every per 1-SD increase in PhenoAge acceleration, the HRs (95% CI) were 1.18 (1.17, 1.19) for FCRMD, 1.37 (1.35, 1.39) for CRMM, and 1.27 (1.26, 1.28) for all-cause death. Similarly, KDMAge acceleration was positively associated with the risks of FCRMD, CRMM, and all-cause death.

**Figure 2:**
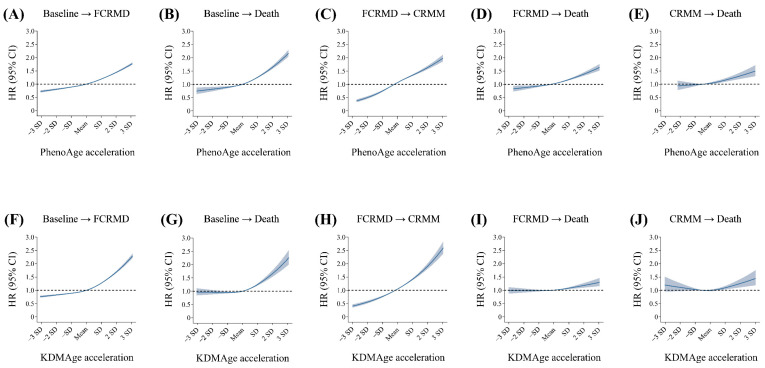
Dose–response curves of biological age acceleration with different transitions in pattern A using the restricted cubic spline multi-state model. (**A**–**E**) Associations of PhenoAge acceleration with the risk of transitions from baseline to FCRMD (**A**); from baseline to death (**B**); from FCRMD to CRMM (**C**); from FCRMD to death (**D**); and from CRMM to death (**E**). (**F**–**J**). The associations of KDMAge acceleration with the risk of transitions from baseline to FCRMD (**F**); from baseline to death (**G**); from FCRMD to CRMM (**H**); from FCRMD to death (**I**); and from CRMM to death (**J**). Estimates are presented per SD increase. Models were adjusted for age, sex, ethnicity, Townsend deprivation index, education, BMI, smoking status, alcohol consumption, physical activity, and dietary behaviors. Abbreviations: FCRMD: first cardio-renal–metabolic disease; CRMM: cardio-renal–metabolic multimorbidity (the coexistence of two or three CRMDs); PhenoAge: phenotypic age; KDMAge: Klemera–Doubal method age.

Multi-state analysis showed that BA acceleration was associated with all five transitions in CRMM progression (Table 2). Fully adjusted HRs (95% CI) per 1-SD increase in PhenoAge acceleration were 1.18 (1.17, 1.19) for baseline to FCRMD; 1.24 (1.22, 1.26) for FCRMD to CRMM; 1.25 (1.22, 1.27) for baseline to death; 1.13 (1.11, 1.15) for FCRMD to death; and 1.09 (1.06, 1.12) for CRMM to death. KDMAge acceleration showed positive associations across all five transitions, though the association with progression from CRMM to death was weaker (HR [95% CI]: 1.04 [1.00, 1.08]). Further analysis showed that participants with above-mean BA acceleration showed a dose–response relationship across all five transitions (Figure 2). Associations remained consistent when analyzed per IQR increase (Figure S3), except for the transition from CRMM to death for KDMAge acceleration, which was not significant (HR [95% CI]: 1.01 [0.97, 1.06]).

Further analysis using the multi-state model divided FCRMD into T2DM, CVD, and CKD (Table 3), indicating that a per 1-SD increase in PhenoAge acceleration was associated with all eleven transitions. A per 1-SD increase in KDMAge acceleration was associated with most transitions but not with certain transitions related to death, including the transition from T2DM to death (HR [95% CI]: 0.96 [0.88, 1.05]), from CKD to death (HR [95% CI]: 0.94 [0.86, 1.04]), and from CRMM to death (HR [95% CI]: 1.03 [0.99, 1.07]). When BA acceleration was dichotomized, the results remained largely unchanged. For PhenoAge acceleration, compared with biologically younger individuals, biologically older individuals showed higher risks across all eleven transitions, except for the transition from CKD to death (HR [95% CI]: 1.27 [0.97, 1.65]), possibly due to a limited sample size. For KDMAge acceleration, most transitions showed higher risks, including the transition from CRMM to death; however, the transitions from T2DM to death (HR [95% CI]: 0.97 [0.81, 1.17]) and from CKD to death (HR [95% CI]: 0.85 [0.70, 1.05]) lacked statistical significance. Furthermore, in the disease progression analysis of CRMM (transition pattern C, Figure S4), BA acceleration was associated with all transitions, except for the transitions from T2DM and CKD to three CRMMs for PhenoAge acceleration, potentially due to a small sample size.

### 3.3. BA Acceleration and Life Expectancy

**Figure 3:**
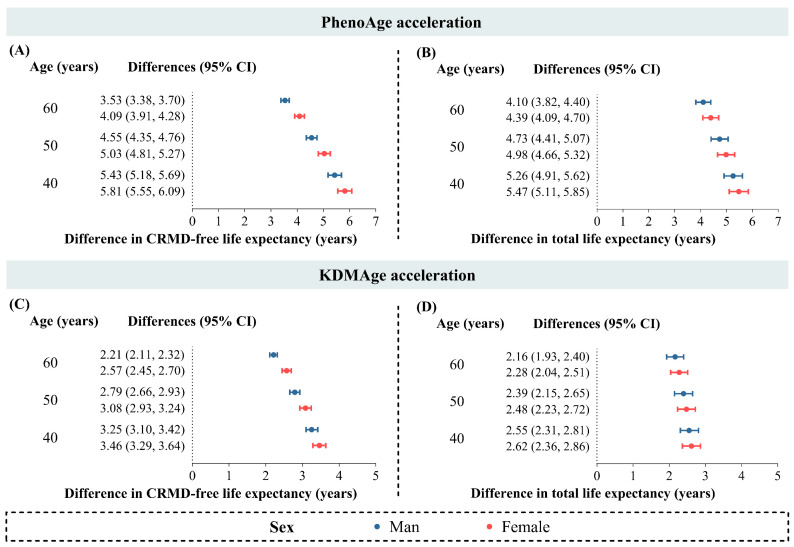
Life expectancy differences between biologically older and younger individuals. Being biologically older is defined as a PhenoAge or KDMAge acceleration > 0. Being biologically younger is defined as an acceleration ≤ 0. (**A**,**B**) Life expectancy for biologically older vs. younger individuals by PhenoAge acceleration: (**A**) CRMD-free life expectancy; (**B**) total life expectancy. (**C**,**D**) Life expectancy for biologically older vs. younger individuals by KDMAge acceleration: (**C**) CRMD-free life expectancy; (**D**) total life expectancy. Predictions for ages 40, 50, and 60 without CRMD using multi-state models stratified by sex. Models were adjusted for ethnicity, Townsend deprivation index, education, BMI, smoking, alcohol, physical activity, and diet.

Both males and females demonstrated reductions in CRMD-free and total life expectancy associated with higher BA acceleration, with PhenoAge acceleration linked to greater losses (Figure 3). Among the males classified as biologically older based on PhenoAge acceleration compared with biologically younger males, CRMD-free life expectancy was associated with a reduction (3.53 [3.38, 3.70] years at age 60 and 5.43 [5.18, 5.69] years at age 40) and total life expectancy was associated with a reduction (4.10 [3.82, 4.40] years at age 60 and 5.26 [4.91, 5.62] years at age 40). Similarly, among males classified as biologically older based on KDMAge acceleration compared with biologically younger males, CRMD-free life expectancy was associated with a reduction (2.21 [2.11, 2.32] years at age 60 and 3.25 [3.10, 3.42] years at age 40) and total life expectancy was associated with a reduction (2.16 [1.93, 2.40] years at age 60 and 2.55 [2.31, 2.81] years at age 40). The same pattern was observed in females.

### 3.4. Subgroup Analyses

We observed modification effects using sociodemographic characteristics (age, economic status, education) and lifestyle factors (smoking status, alcohol consumption, physical activity, diet) on the relationship between BA acceleration and one or more transitions (Tables S6–S14). When exposed to the same increase in BA acceleration, older individuals faced a higher risk of progressing from FCRMD to CRMM; healthy individuals with low economic status, low education, non-moderate alcohol consumption, low physical activity, or unhealthy dietary behaviors demonstrated an elevated risk of developing FCRMD; smoking history modified the transitions from baseline to FCRMD, baseline to death, and FCRMD to death. Findings from the multiplicative interaction model were consistent with those of the stratified analysis (Tables S6–S14). Specifically, older age, low physical activity, smoking history, and unhealthy dietary behaviors showed a synergistic effect, whereas moderate alcohol consumption, high economic status, and high education exhibited an antagonistic effect.

### 3.5. Sensitivity Analyses

The sensitivity analysis results are summarized in Table S15 and Figure S5. The PhenoAge acceleration results remained stable, but KDMAge acceleration results lost statistical significance in transitions from CRMM to death when excluding those diagnosed with any CRMD within two years after enrollment or participants with abnormal baseline levels of glucose, glycated hemoglobin, eGFR, or albuminuria.

## 4. Discussion

### 4.1. Principal Findings

Using prospective cohort data from approximately 270,000 UK Biobank adults, we first examined how accelerated biological aging, measured by two novel integrative BA algorithms (PhenoAge and KDMAge), was related to the progression from health to FCRMD and then to CRMM and death. We found that BA acceleration was associated with all five transitions, with PhenoAge acceleration showing stronger associations. After splitting FCRMD into T2DM, CVD, and CKD, PhenoAge acceleration consistently exhibited stronger associations. Additionally, biologically older individuals were correlated with reduced CRMD-free and total life expectancy at ages 40, 50, and 60, with larger reductions in those defined as biologically older by PhenoAge acceleration. We also identified subgroups more vulnerable to BA acceleration in certain transitions.

### 4.2. Comparison with Previous Studies

Consistent with prior studies, we found that higher BA acceleration was correlated with single CRMDs, including T2DM [21,22], CVD [22,23,24], and CKD [25]. For example, a Korean study found that BA acceleration, calculated using principal component analysis and based on clinical parameters, was associated with the incidence of diabetes, heart disease, and stroke [22]. A UK Biobank study showed that individuals with higher PhenoAge or KDMAge acceleration had an increased risk of CKD, independent of CA [25]. T2DM, CVD, and CKD share pathophysiological mechanisms, often co-occur, and are proposed to be managed as a syndrome through unified prevention and treatment [1]. However, to our knowledge, no study has yet explored the temporal association between BA acceleration and the progression from a single CRMD to CRMM and death. The multi-state model effectively accounts for various disease stages and competing risks [29,32,33], supporting the dynamic monitoring of progression within a single framework.

In this study, we employed a multi-state model to assess, for the first time, the association between BA acceleration and the risk of the subsequent progression of a single CRMD. We found that baseline BA acceleration was correlated with an increased risk of transition from FCRMD to CRMM. Our findings are consistent with evidence linking BA acceleration to cardio-renal–metabolic health. For instance, a prospective cohort study indicated that patients with diabetes have a higher risk of progressing to ischemic heart disease or stroke [29]. Another study found that in CKD patients, biological aging—measured by telomere length, KDMAge, and PhenoAge—was associated with an elevated risk of CVD [40]. We further categorized CRMM into two coexisting CRMDs (two CRMMs) and three coexisting CRMDs (three CRMMs), revealing that BA acceleration was associated with the progression from a single specific CRMD to two specific CRMMs and three CRMMs, thus extending existing research. The scarcity of comparable studies may stem from two main factors: first, although the interplay between T2DM, CVD, and CKD is well-recognized, their integration as a unified syndrome is a recent concept [1]; second, CRMD patients often face poor prognosis and high mortality [8].

Furthermore, our study showed BA acceleration to be associated with mortality risk in participants with or without CRMD. PhenoAge acceleration was associated with increased risk across all mortality-related transitions, whereas KDMAge acceleration showed no significant association with certain transitions, such as those from T2DM or CKD to death. Additionally, individuals defined as biologically older by PhenoAge were linked to a greater loss in life expectancy. These differences might partly stem from the selection of biomarkers and their degrees of contribution: PhenoAge primarily focuses on biomarkers of inflammation and immune function [25,29], potentially capturing a multi-system decline in disease progression more comprehensively. In CKD patients [41] and T2DM patients [42], inflammation levels have served as an independent predictor of mortality risk. By comparison, KDMAge concentrates on heart and lung function [25,29]. While it accurately represents cardiovascular and respiratory health, it is less sensitive to inflammation and other significant pathological processes. In addition, KDMAge only tracks CA with a linear increase [30,43], while PhenoAge captures both CA and mortality risk with an exponential increase [30,43], allowing it to capture nonlinear increases in mortality risk. This might account for PhenoAge’s greater sensitivity to mortality risk and lifespan loss. Our findings offer important implications for clinical practice. The blood, biochemical, and clinical parameters used for the BA assessment can be easily obtained in clinical environments, allowing for the timely identification and prioritized monitoring of high-risk individuals with higher BA acceleration. Our results show that higher PhenoAge acceleration is more closely associated with CRMM progression risk, warranting the prioritization of secondary prevention measures. If interventions (e.g., active lifestyles or aging-targeted drugs) improve clinical parameters in BA algorithms, this might lead to the prevention, control, and delay of age-related CRMM progression and healthy lifespans might be extended.

We identified subgroups more susceptible to BA acceleration. Specifically, FCRMD patients aged 65 years or older were more vulnerable to progressing to CRMM. This may be due to the poorer health of older populations [44,45]. Healthy individuals at baseline with unhealthy lifestyle factors (e.g., non-moderate alcohol consumption, low physical activity, or unhealthy dietary behaviors) were found to face a higher risk of developing FCRMD. This was likely associated with the cumulative impact of long-term unhealthy behaviors on metabolic disorders [46]. Moreover, BA acceleration was more closely linked to the transition from health to FCRMD among individuals with lower socioeconomic status or education, possibly associated with limited healthcare access [47,48] and increased exposure to stressful social conditions [49]. Notably, smoking history modified the most transitions, including those from health to FCRMD, health to death, and FCRMD to death. This aligns with a study showing how smoking is most strongly associated with CRMM progression among seven lifestyle factors [2]. This underscores the urgent need for smoking control measures to prevent CRMM progression. More research is needed to explore how biological aging and sociodemographic and lifestyle factors contribute to CRMM progression.

### 4.3. Mechanism

Clinical parameter-based BA may affect CRMM progression through interconnected mechanisms. Accelerated biological aging is often associated with altered adipokine expression [16,17], while dysregulated lipid metabolism may drive CRMD onset and subsequent progression [50]. BA acceleration is associated with the activation of pro-inflammatory [18] and pro-oxidative pathways [19]. These disruptions in inflammation and oxidative stress are associated with increased pro-fibrotic factor production, which may lead to pathological changes like tubulointerstitial fibrosis and glomerulosclerosis, which are key to CKD initiation [51,52]. Furthermore, inflammation is linked to atherosclerosis, endothelial damage, and myocardial injury [53,54], which correlate with CVD and mortality risks. Additionally, BA acceleration is linked to insulin resistance [20], which may contribute to a harmful feedback loop between T2DM, CVD, and CKD [50]. Further research is needed to clarify these complex mechanisms.

### 4.4. Strengths and Limitations

The key strengths of this study include the use of the multi-state model, data from a large, prospective cohort, and BA algorithms with the potential for broader clinical application. Nevertheless, several limitations warrant consideration. First, biomarkers in the BA algorithms were measured only at baseline, limiting our ability to track BA acceleration over time; however, relying on baseline BA acceleration reduces reverse causality after the onset of outcomes. Second, the association between BA acceleration and CRMM progression might be related to cardio-renal–metabolic health biomarkers incorporated into the algorithms; however, this association was observed even after excluding these biomarkers. Third, our study population, primarily of European descent, might have introduced healthy volunteer bias. Participants generally presented better health, higher education, and socioeconomic status than other cohorts, which limited the generalizability of our findings. Caution is advised when applying these results to broader populations. Future studies should validate these findings in more diverse and representative groups. Fourth, our analyses were conducted in a general population, and the clinical implementation of BA acceleration still requires further study. Finally, the observational nature of this study limits the ability to establish causality.

## 5. Conclusions

Our study demonstrated how BA acceleration, particularly PhenoAge acceleration, is associated with an increased risk of transitions related to CRMM progression and a marked reduction in life expectancy. BA acceleration can guide interventions throughout CRMM progression and identify risks for specific transitions by integrating sociodemographic and lifestyle factors.

## Figures and Tables

**Table 1:** Baseline characteristics of 278,927 participants by incident disease states.

Characteristics ^a^	Total (*n* = 278,927)	Free of CRMD (*n* = 214,834)	Incident FCRMD (*n* = 54,581)	Incident CRMM (*n* = 9512)
Age (years)	55.69 (8.10)	54.62 (8.02)	58.98 (7.35)	60.93 (6.73)
Sex (male)	122,796 (44.0)	88,785 (41.3)	28,753 (52.7)	5258 (55.3)
Ethnicity (White)	265,381 (95.1)	204,287 (95.1)	52,184 (95.6)	8910 (93.7)
Townsend deprivation index (>median)	139,730 (50.1)	106,664 (49.6)	27,728 (50.8)	5338 (56.1)
Educational level (high)	136,707 (49.0)	109,100 (50.8)	24,189 (44.3)	3418 (35.9)
Body mass index (kg·m^−2^)	27.01(4.50)	26.67 (4.32)	27.88 (4.73)	29.86 (5.34)
Smoking status				
Never	158,072 (56.7)	125,926 (58.6)	27,839 (51.0)	4307 (45.3)
Former	92,457 (33.1)	68,569 (31.9)	20,122 (36.9)	3766 (39.6)
Current	28,398 (10.2)	20,339 (9.5)	6620 (12.1)	1439 (15.1)
Alcohol consumption				
None	64,576 (23.1)	47,707 (22.2)	13,728 (25.1)	3141 (33.0)
Moderate	139,097 (49.9)	108,772 (50.6)	26,125 (47.9)	4200 (44.2)
Heavy	75,254 (27.0)	58,355 (27.2)	14,728 (27.0)	2171 (22.8)
Physical activity				
Low	49,745 (17.8)	37,573 (17.5)	10,068 (18.4)	2104(22.1)
Moderate	113,783(40.8)	88,041 (41.0)	21,877 (40.1)	3865 (40.7)
High	115,399(41.4)	89,220 (41.5)	22,636 (41.5)	3543 (37.2)
Dietary behaviors (healthy)	45,823 (16.4)	35,098 (16.3)	9307 (17.1)	1418 (14.9)
Biological age measures				
PhenoAge (years)	49.18 (9.07)	47.84 (8.79)	53.12 (8.41)	56.92 (8.34)
PhenoAge acceleration (years)	−6.51 (4.26)	−6.78 (4.09)	−5.86 (4.50)	−4.01 (5.26)
KDMAge (years)	52.38 (12.41)	50.82 (12.08)	56.69 (11.87)	62.92 (11.88)
KDMAge acceleration (years)	−3.31 (9.76)	−3.80 (9.48)	−2.29 (10.27)	2.00 (10.86)
Components of biological age measures				
Lymphocyte (%) ^b^	29.15 (7.36)	29.30 (7.28)	28.74 (7.59)	28.22 (7.71)
Mean cell volume (fL) ^b^	82.82 (5.26)	82.78 (5.20)	83.03 (5.43)	82.60 (5.49)
Serum glucose (mmol/L) ^b^	4.99 (0.84)	4.95 (0.72)	5.09 (1.04)	5.45 (1.68)
Red cell distribution width (%) ^b^	13.45 (0.94)	13.42 (0.94)	13.51 (0.94)	13.64 (1.05)
White blood cell count (1000 cells/uL) ^b^	6.78 (1.86)	6.71 (1.79)	6.96 (2.02)	7.39 (2.10)
Albumin (g/dL) ^b,c^	4.53 (0.26)	4.54 (0.26)	4.50 (0.26)	4.47 (0.27)
Creatinine (mg/dL) ^b,c^	0.81 (0.16)	0.80 (0.15)	0.83 (0.17)	0.90 (0.23)
*C*-reactive protein (mg/dL) ^b,c^	0.24 (0.40)	0.22 (0.37)	0.29 (0.45)	0.40 (0.54)
Alkaline phosphatase (U/L) ^b,c^	82.53 (25.25)	81.37 (24.50)	85.74 (27.00)	90.19 (28.40)
FEV1 (L) **^c^**	2.77 (0.78)	2.81 (0.77)	2.70 (0.79)	2.51 (0.76)
SBP (mm Hg) **^c^**	137.15 (18.20)	135.53 (17.80)	142.02 (18.36)	145.86 (18.60)
Total cholesterol (mg/dL) **^c^**	225.41 (41.79)	225.21 (41.30)	226.77 (42.99)	222.24 (45.34)
Glycated hemoglobin (%) **^c^**	5.36 (0.43)	5.32 (0.36)	5.45 (0.52)	5.75 (0.79)
Blood urea nitrogen (mg/dL) **^c^**	14.90 (3.52)	14.71 (3.38)	15.35 (3.69)	16.54 (4.62)

^a^ Continuous variables are presented as the mean (SD), and categorical variables as the number (%). ^b^ Used in the construction of PhenoAge. **^c^** Used in the construction of KDMAge. Abbreviations: CRMD: cardio-renal–metabolic disease; FCRMD: first cardio-renal–metabolic disease; CRMM: cardio-renal–metabolic multimorbidity (the coexistence of two or three CRMDs); PhenoAge: phenotypic age; KDMAge: Klemera–Doubal method age; FEV1: forced expiratory volume in one second; SBP: systolic blood pressure.

**Table 2:** The associations of biological age acceleration with different transitions in pattern A using the multi-state model.

Transition	Cases	PhenoAge Acceleration			KDMAge Acceleration		
		Biologically Younger	Biologically Older	Per 1 SD	Biologically Younger	Biologically Older	Per 1 SD
Baseline to FCRMD	64,093	Reference	1.64 (1.59, 1.68)	1.18 (1.17, 1.19)	Reference	1.37 (1.35, 1.39)	1.22 (1.21, 1.23)
Baseline to death	7172	Reference	1.95 (1.80, 2.10)	1.25 (1.22, 1.27)	Reference	1.24 (1.18, 1.30)	1.16 (1.13, 1.19)
FCRMD to CRMM	9512	Reference	1.67 (1.58, 1.77)	1.24 (1.22, 1.26)	Reference	1.55 (1.49, 1.62)	1.33 (1.30, 1.35)
FCRMD to death	8701	Reference	1.42 (1.34, 1.51)	1.13 (1.11, 1.15)	Reference	1.06 (1.02, 1.11)	1.05 (1.02, 1.07)
CRMM to death	2192	Reference	1.33 (1.20, 1.47)	1.09 (1.06, 1.12)	Reference	1.11 (1.01, 1.21)	1.04 (1.00, 1.08)

Models were adjusted for age, sex, ethnicity, Townsend deprivation index, education, BMI, smoking status, alcohol consumption, physical activity, and dietary behavior. Abbreviations: FCRMD: first cardio-renal–metabolic disease; CRMM: cardio-renal–metabolic multimorbidity (the coexistence of two or three CRMDs); PhenoAge: phenotypic age; KDMAge: Klemera–Doubal method age; SD: standard deviation.

**Table 3:** The associations of biological age acceleration with different transitions in pattern B using the multi-state model.

Transition	Cases	PhenoAge Acceleration			KDMAge Acceleration		
		Biologically Younger	Biologically Older	Per 1 SD	Biologically Younger	Biologically Older	Per 1 SD
Baseline to T2DM	8171	Reference	1.87 (1.75, 1.99)	1.27 (1.24, 1.29)	Reference	1.92 (1.83, 2.01)	1.50 (1.47, 1.53)
Baseline to CVD	47,794	Reference	1.41 (1.36, 1.45)	1.11 (1.10, 1.12)	Reference	1.23 (1.21, 1.26)	1.13 (1.12, 1.14)
Baseline to CKD	5981	Reference	2.77 (2.58, 2.98)	1.40 (1.38, 1.42)	Reference	1.88 (1.78, 1.98)	1.59 (1.55, 1.63)
Baseline to death	7172	Reference	1.97 (1.83, 2.12)	1.25 (1.23, 1.27)	Reference	1.25 (1.19, 1.31)	1.17 (1.14, 1.20)
T2DM to CRMM	1804	Reference	1.31 (1.16, 1.47)	1.14 (1.10, 1.19)	Reference	1.20 (1.09, 1.32)	1.15 (1.10, 1.20)
T2DM to death	478	Reference	1.50 (1.20, 1.88)	1.10 (1.02, 1.18)	Reference	0.97 (0.81, 1.17)	0.96 (0.88, 1.05)
CVD to CRMM	4072	Reference	1.71 (1.56, 1.86)	1.26 (1.23, 1.29)	Reference	1.60 (1.49, 1.70)	1.34 (1.30, 1.38)
CVD to death	7838	Reference	1.57 (1.47, 1.68)	1.17 (1.15, 1.20)	Reference	1.15 (1.10, 1.21)	1.10 (1.08, 1.13)
CKD to CRMM	1489	Reference	1.58 (1.39, 1.80)	1.15 (1.10, 1.20)	Reference	1.28 (1.15, 1.42)	1.20 (1.15, 1.26)
CKD to death	385	Reference	1.27 (0.97, 1.65)	1.12 (1.03, 1.22)	Reference	0.85 (0.70, 1.05)	0.94 (0.86, 1.04)
CRMM to death	8171	Reference	1.29 (1.15, 1.44)	1.07 (1.04, 1.11)	Reference	1.13 (1.02, 1.24)	1.03 (0.99, 1.07)

Models were adjusted for age, sex, ethnicity, Townsend deprivation index, education, BMI, smoking status, alcohol consumption, physical activity, and dietary behaviors. Abbreviations: CRMM: cardio-renal–metabolic multimorbidity (the coexistence of two or three CRMDs); T2DM: type 2 diabetes mellitus; CVD: cardiovascular disease; CKD: chronic kidney disease; PhenoAge: phenotypic age; KDMAge: Klemera–Doubal method age.

## Data Availability

The availability of this data is restricted. The data were sourced from the UK Biobank and, with permission from UK Biobank, can be accessed at https://www.ukbiobank.ac.uk.

## Supplementary Materials

The following supporting information can be downloaded at: https://www.mdpi.com/article/10.3390/nu17111783/s1, Figure S1: Flowchart of participant selection; Figure S2: Numbers (percentages) of participants from baseline to specific FCRMD, then to specific two CRMM and three CRMM; Figure S3: Associations of biological age acceleration per IQR increase with the risks of FCRMD, CRMM, and death of pattern A using the multi-state model; Figure S4: Associations of biological age acceleration with the risks of specific FCRMD, specific two CRMM, and three CRMM of pattern C using the multi-state model; Figure S5: Associations of biological age acceleration with the risks of FCRMD, CRMM, and death of pattern A using the multi-state model after removing specific biomarkers in the construction of biological age algorithms; Table S1: Detailed definitions of incident outcomes in this study; Table S2: Missing information on components of biological age algorithms; Table S3: Components of recent dietary recommendations for cardiovascular health in this study; Table S4: Missing information on covariates; Table S5: Associations of biological age acceleration with the risks of FCRMD, CRMM, and all-cause death using the Cox regression model; Table S6: Associations of the biological age acceleration with the trajectories of cardio-renal-metabolic multimorbidity of pattern A using the multi-state model, stratified by age groups; Table S7: Associations of the biological age acceleration with the trajectories of cardio-renal-metabolic multimorbidity of pattern A using the multi-state model, stratified by sex; Table S8: Associations of biological age acceleration with the trajectories of cardio-renal-metabolic multimorbidity of pattern A using the multi-state model, stratified by Townsend deprivation index; Table S9: Associations of biological age acceleration with the trajectories of cardio-renal-metabolic multimorbidity of pattern A using the multi-state model, stratified by education level; Table S10: Associations of biological age acceleration with the trajectories of cardio-renal-metabolic multimorbidity of pattern A using the multi-state model, stratified by BMI; Table S11: Associations of biological age acceleration with the trajectories of cardio-renal-metabolic multimorbidity of pattern A using the multi-state model, stratified by smoking status; Table S12: Associations of biological age acceleration with the trajectories of cardio-renal-metabolic multimorbidity of pattern A using the multi-state model, stratified by alcohol consumption; Table S13: Associations of biological age acceleration with the trajectories of cardio-renal-metabolic multimorbidity of pattern A using the multi-state model, stratified by physical activity; Table S14: Associations of biological age acceleration with the trajectories of cardio-renal-metabolic multimorbidity of pattern A using the multi-state model, stratified by dietary behaviors; Table S15: Sensitivity analysis of associations between biological age acceleration and trajectories of cardio-renal-metabolic multimorbidity.

## Abbreviations

BA: Biological Age; CRMDs: Cardio-Renal–Metabolic Diseases; CRMM: Cardio-Renal–Metabolic Multimorbidity; FCRMD: First Cardio-Renal–Metabolic Disease; PhenoAge: Phenotypic Age; KDMAge: Klemera–Doubal Method Age; CA: Chronological Age; HR: Hazard Ratio; SD: Standard Deviation; CI: Confidence Interval; T2DM: Type 2 Diabetes Mellitus; CVD: Cardiovascular Disease; CKD: Chronic Kidney Disease; FEV1: Forced Expiratory Volume in One Second; SBP: Systolic Blood Pressure; NHANES: National Health and Nutrition Examination Survey; BMI: Body Mass Index; IPAQ: International Physical Activity Questionnaire; MET: Metabolic Equivalent of Task; eGFR: Estimated Glomerular Filtration Rate; IQR: Interquartile Range.
