# Supplementary material for: Biological Age Acceleration Associated with the Progression Trajectory of Cardio-Renal–Metabolic Multimorbidity: A Prospective Cohort Study
Source: Nutrients. 2025 May 24;17(11):1783. doi: 10.3390/nu17111783 (PMC12157706; doi:10.3390/nu17111783)
Supplement: Supplementary file 1 [file nutrients-17-01783-s001.zip › nutrients-3613594-supplementary.pdf]

Supplementary Materials

Biological Age Acceleration Associated with the Progression Trajectory of Cardio-Renal–Metabolic Multimorbidity: A Prospective Cohort Study

- **Supplementary figures**

Figure S1: Flowchart of participant selection

Figure S2: Numbers (percentages) of participants from baseline to specific FCRMD, then to specific two CRMM and three CRMM

Figure S3: Associations of biological age acceleration per IQR increase with the risks of FCRMD, CRMM, and death of pattern A using the multi-state model

Figure S4: Associations of biological age acceleration with the risks of specific FCRMD, specific two CRMM, and three CRMM of pattern C using the multi-state model

Figure S5: Associations of biological age acceleration with the risks of FCRMD, CRMM, and death of pattern A using the multi-state model after removing specific biomarkers in the construction of biological age algorithms

- **Supplementary tables**

Table S1: Detailed definitions of incident outcomes in this study

Table S2: Missing information on components of biological age algorithms

Table S3: Components of recent dietary recommendations for cardiovascular health in this study

Table S4: Missing information on covariates

Table S5: Associations of biological age acceleration with the risks of FCRMD, CRMM, and all-cause death using the Cox regression model

Table S6: Associations of the biological age acceleration with the trajectories of cardio-renal-metabolic multimorbidity of pattern A using the multi-state model, stratified by age groups

Table S7: Associations of the biological age acceleration with the trajectories of cardio-renal-metabolic multimorbidity of pattern A using the multi-state model, stratified by sex

Table S8: Associations of biological age acceleration with the trajectories of cardio-renal-metabolic multimorbidity of pattern A using the multi-state model, stratified by Townsend deprivation index

Table S9: Associations of biological age acceleration with the trajectories of cardio-renal-metabolic multimorbidity of pattern A using the multi-state model, stratified by education level

Table S10: Associations of biological age acceleration with the trajectories of cardio-renal-metabolic multimorbidity of pattern A using the multi-state model, stratified by BMI

Table S11: Associations of biological age acceleration with the trajectories of cardio-renal-metabolic multimorbidity of pattern A using the multi-state model, stratified by smoking status

Table S12: Associations of biological age acceleration with the trajectories of cardio-renal-metabolic multimorbidity of pattern A using the multi-state model, stratified by alcohol consumption

Table S13: Associations of biological age acceleration with the trajectories of cardio-renal-metabolic multimorbidity of pattern A using the multi-state model, stratified by physical activity

Table S14: Associations of biological age acceleration with the trajectories of cardio-renal-metabolic multimorbidity of pattern A using the multi-state model, stratified by dietary behaviors

Table S15: Sensitivity analysis of associations between biological age acceleration and trajectories of cardio-renal-metabolic multimorbidity

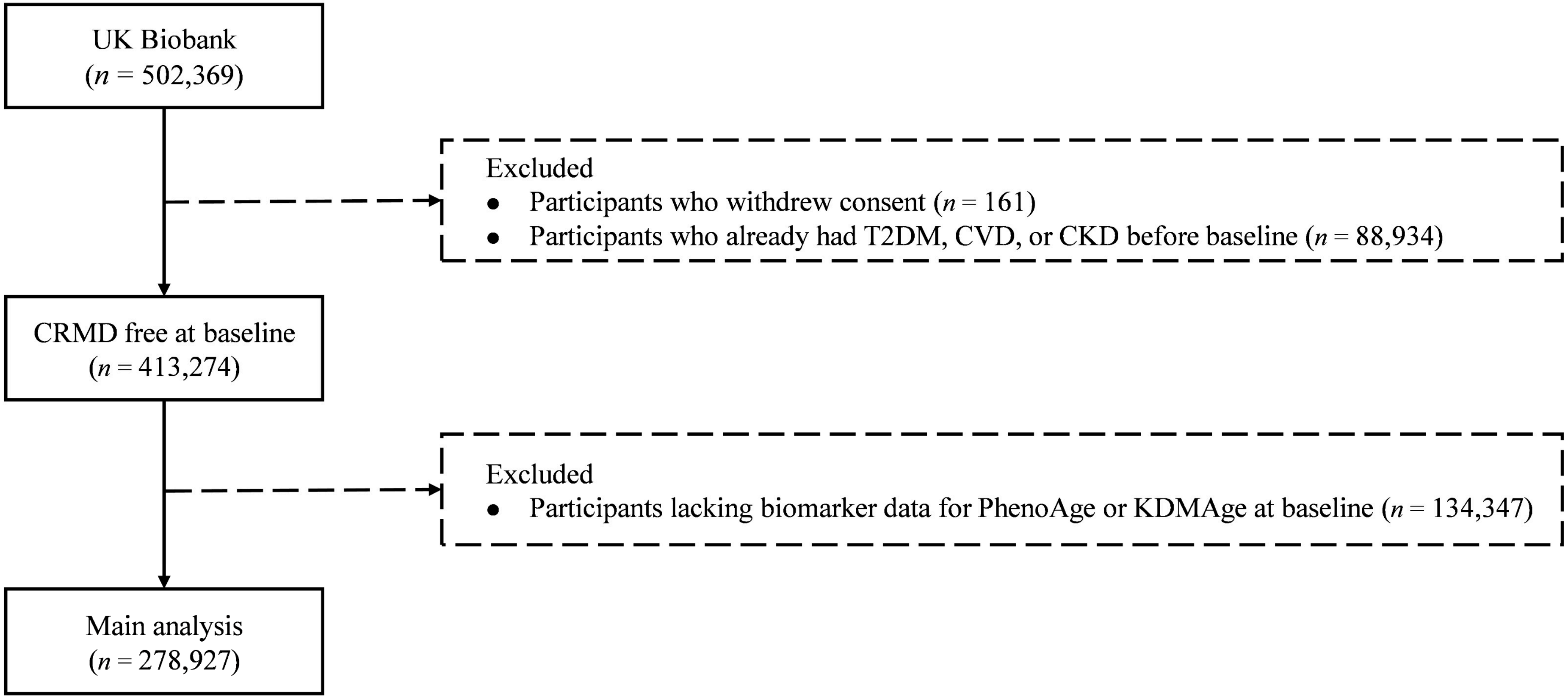

Figure S1: Flowchart of participant selection

Abbreviations: CRMD: cardio-renal-metabolic disease; T2DM: type 2 diabetes mellitus; CVD: cardiovascular disease; CKD: chronic kidney disease; PhenoAge: phenotypic age; KDMAge: Klemera-Doubal method age.

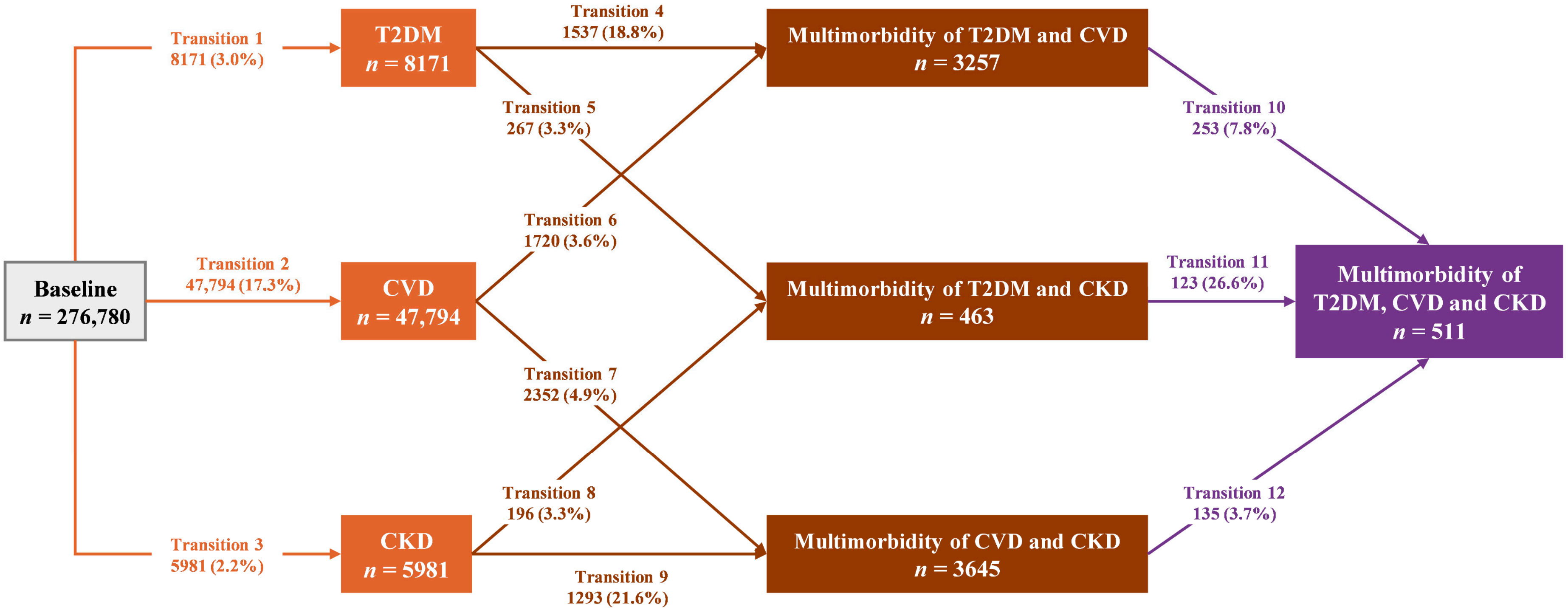

Figure S2: Numbers (percentages) of participants from baseline to specific FCRMD, then to specific two CRMM and three CRMM

Abbreviations: FCRMD: first cardio-renal-metabolic disease; CRMM: cardio-renal-metabolic multimorbidity (the coexistence of two or three CRMDs); T2DM: type 2 diabetes mellitus; CVD: cardiovascular disease; CKD: chronic kidney disease.

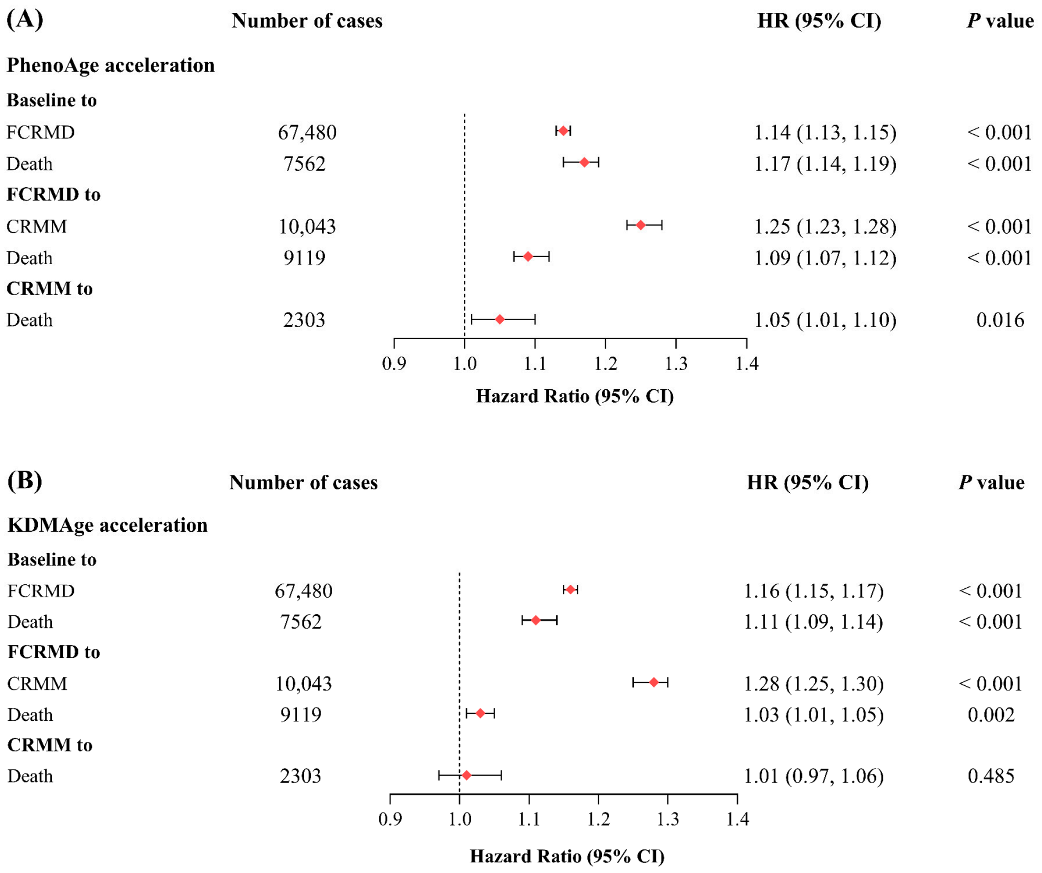

Figure S3: Associations of biological age acceleration per IQR increase with the risks of FCRMD, CRMM, and death of pattern A using the multi-state model

(A) Associations of PhenoAge acceleration per IQR increase with five transitions in the CRMM progression trajectory; (B) Associations of KDMAge acceleration per IQR increase with five transitions in the CRMM progression trajectory.

Estimates are presented per SD increase.

Models were adjusted for age, sex, ethnicity, Townsend deprivation index, education, BMI, smoking status, alcohol consumption, physical activity, and dietary behaviors.

Abbreviations: FCRMD: first cardio-renal-metabolic disease; CRMM: cardio-renal-metabolic multimorbidity (the coexistence of two or three CRMDs); PhenoAge: phenotypic age; KDMAge: Klemera-Doubal method age; HR: hazard ratio; CI: confidence interval; IQR: interquartile range.

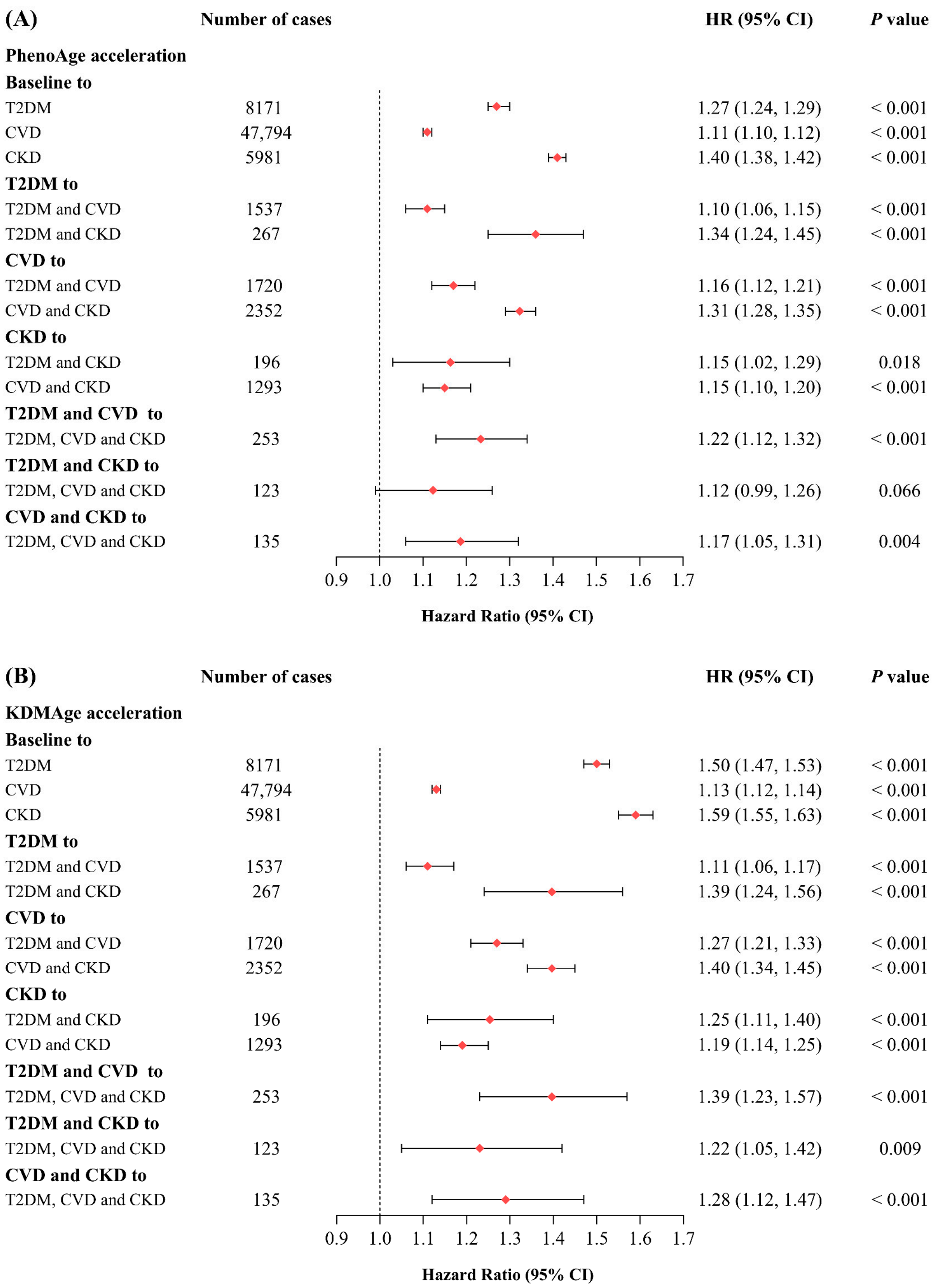

Figure S4: Associations of biological age acceleration with the risks of specific FCRMD, specific two CRMM, and three CRMM of pattern C using the multi-state model

(A) Associations of PhenoAge acceleration with twelve transitions of CRMM disease progression;
(B) Associations of KDMAge acceleration with twelve transitions of CRMM disease progression.
Estimates are presented per SD increase.
Models were adjusted for age, sex, ethnicity, Townsend deprivation index, education, BMI, smoking status, alcohol consumption, physical activity, and dietary behaviors.
Abbreviations: FCRMD: first cardio-renal-metabolic disease; CRMM: cardio-renal-metabolic multimorbidity (the coexistence of two or three CRMDs); T2DM: type 2 diabetes mellitus; CVD: cardiovascular disease; CKD: chronic kidney disease; PhenoAge: phenotypic age; KDMAge: Klemera-Doubal method age; HR: hazard ratio; CI: confidence interval.

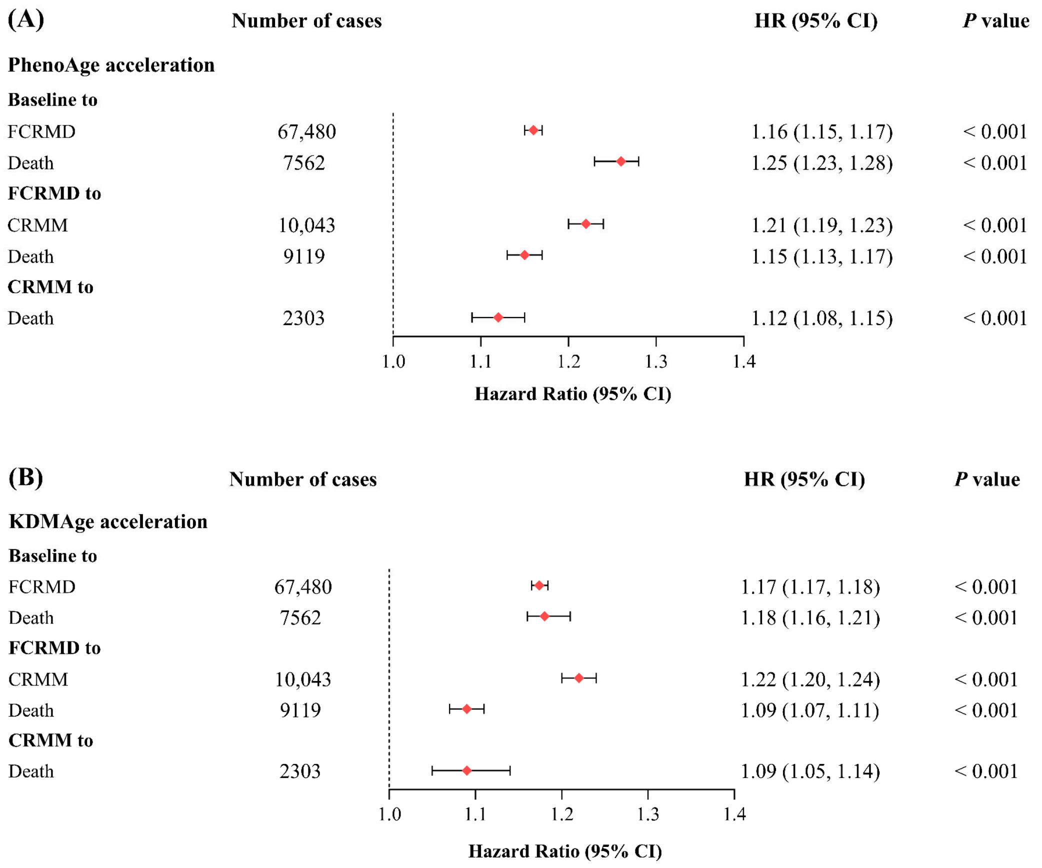

Figure S5: Associations of biological age acceleration with the risks of FCRMD, CRMM, and death of pattern A using the multi-state model after removing specific biomarkers in the construction of biological age algorithms

(A) Associations of PhenoAge acceleration with five transitions in the CRMM progression trajectory after removing creatinine and serum glucose; (B) Associations of KDMAge acceleration with five transitions in the CRMM progression trajectory after removing glycated hemoglobin.

Estimates are presented per SD increase.
Models were adjusted for age, sex, ethnicity, Townsend deprivation index, education, BMI, smoking status, alcohol consumption, physical activity, and dietary behaviors.
Abbreviations: FCRMD: first cardio-renal-metabolic disease; CRMM: cardio-renal-metabolic multimorbidity (the coexistence of two or three CRMDs); PhenoAge: phenotypic age; KDMAge: Klemera-Doubal method age; HR: hazard ratio; CI: confidence interval.

Table S1: Detailed definitions of incident outcomes in this study

Diagnosis	Field IDs	Codes
	➢ Non-cancer illness, self-report (20002)	1223
	➢ First occurrences (130708, 130709); Summary Diagnosis, ICD10 (41270); Death register (40000,40001,40002)	E11
	➢ Summary Diagnoses, ICD9 (41271)	25000, 25010
	➢ Medications (20003)	1140883066, 1140884600, 1141189090, 1141152590, 1140874744, 1140874718, 1141156984, 1140868902, 1141171646, 1141168660, 1141173882
	➢ Non-cancer illness, self-report (20002)	1067, 1068, 1077, 1078, 1079, 1081, 1082, 1083, 1086, 1088, 1093, 1094, 1426, 1471, 1479, 1483, 1485, 1487, 1488, 1489, 1490, 1491, 1496, 1583, 1584, 1585, 1586, 1587, 1588
CVD	➢ Vascular/heart problems diagnosed by doctor (6150)	1=Heart attack, 2=angina, 3=unspecific stroke
	➢ First occurrences (131056–131057, 131272–131273, 131276–131285,131288–131289, 131296–131379, 131386–131387, 131418–131423); Summary Diagnosis, ICD10 (41270); Death register (40000,40001,40002)	I01, I05-I09, I11, I20-I25, I26-I28, I30-I52, I60-I69, I73, I97-I99, G45

	➢ Summary Diagnoses, ICD9 (41271)	391, 393-398, 410-414, 415-417, 420-429, 430-438, 443, 459
CKD	➢ Non-cancer illness, self-report (20002)	1192, 1193, 1194
	➢ First occurrences (132032–132033, 131290–131291); Summary Diagnosis, ICD10 (41270); Death register (40000,40001,40002)	N18, I12
	➢ Summary Diagnoses, ICD9 (41271)	585
	➢ Summary Operations, OPCS-4	M01

Abbreviations: T2DM: type 2 diabetes mellitus; CVD: cardiovascular disease; CKD: chronic kidney disease; ICD: International Classification of Diseases; OPCS-4: Office of Population Censuses and Surveys Classification of Interventions and Procedures, version 4.

Table S2: Missing information on components of biological age algorithms

Characteristic	Missing number and proportion, *n* (%)
Lymphocyte	20,460 (4.95)
Mean cell volume	26,609 (6.44)
Serum glucose	59,742 (14.46)
Red cell distribution width	19,752 (4.78)
White blood cell count	19,757 (4.78)
Albumin	59,327 (14.36)
Creatinine	26,900 (6.51)
C-reactive protein	27,509 (6.66)
Alkaline phosphatase	26,695 (6.46)
FEV1	35,384 (8.56)
SBP	25,390 (6.14)
Total Cholesterol	26,711 (6.46)
Glycated hemoglobin	29,332 (7.10)
Blood urea nitrogen	26,972 (6.53)
Combinations	134,347 (32.51)

Abbreviations: FEV1: forced expiratory volume in one second; SBP: systolic blood pressure.

Table S3: Components of recent dietary recommendations for cardiovascular health in this study

Diet component	Field IDs	Intake goal
Fruit	fresh fruit (1309)<br>dried fruit (1319)	≥3 servings/day
Vegetable	cooked vegetable (1289)<br>salad/raw vegetable (1299)	≥3 servings/day
Whole grains	whole meal/wholegrain bread (1438, 1448)<br>bran/oat/muesli cereal (1458, 1468)	≥3 servings/day
(Shell)fish	oily fish (1329)<br>non-oily fish (1339)	≥2 servings/week
Dairy	cheese (1408)<br>milk (1418)	≥2 servings/day
Vegetable oils	spread (1428)<br>non-butter spread (2654)<br>bread (1438)	≥2 servings/day
Refined grains	white, brown, other bread (1438, 1448)<br>biscuit, other cereal (1458, 1468)	≤2 servings/day
Processed meats	processed meat (1349)<br>age when last ate meat (3680)	≤1 serving/week
Unprocessed meats	poultry (1359)<br>beef (1369)<br>lamb or mutton (1379)<br>pork (1389)<br>age when last ate meat (3680)	≤2 servings/day
Sugar-sweetened beverages	never consumes drinks containing sugar (6144)	Never drink

Participants meeting intake goals were considered to have adequate intake of the relevant dietary components. A score of 1 was assigned for meeting each goal and 0 for failing to meet it, with the total score ranging from 0 to 10. Healthy dietary habits were defined as meeting at least five intake goals.

Table S4: Missing information on covariates

Characteristic	Missing number and proportion, *n* (%)
Age	0 (0.00)
Sex	0 (0.00)
Ethnicity	1159 (0.42)
Townsend Deprivation Index	334 (0.12)
Educational level	2911 (1.04)
Body mass index	447 (0.16)
Smoking status	1190 (0.43)
Alcohol consumption	631 (0.23)
Physical activity	60,504 (21.69)
Dietary behaviors	21,161 (7.59)
Combinations	77,628 (27.83)

Table S5: Associations of biological age acceleration with the risks of FCRMD, CRMM, and all-cause death using the Cox regression model

Indicator	FCRMD (*n* = 64,093)		CRMM (*n* = 9512)		All-cause death\ (*n* = 18,065)	
	HR (95% CI)	*P* value	HR (95% CI)	*P* value	HR (95% CI)	*P* value
**PhenoAge acceleration**						
Biologically younger	Reference		Reference		Reference	
Biologically older	1.64 (1.59, 1.68)	< 0.001	2.37 (2.24, 2.50)	< 0.001	2.07 (1.99, 2.17)	< 0.001
Per 1 SD	1.18 (1.17, 1.19)	< 0.001	1.37 (1.35, 1.39)	< 0.001	1.27 (1.26, 1.28)	< 0.001
**KDMAge acceleration**						
Biologically younger	Reference		Reference		Reference	
Biologically older	1.37 (1.35, 1.39)	< 0.001	2.01 (1.93, 2.10)	< 0.001	1.37 (1.33, 1.41)	< 0.001
Per 1 SD	1.22 (1.21, 1.23)	< 0.001	1.57 (1.54, 1.61)	< 0.001	1.23 (1.21, 1.25)	< 0.001

Models were adjusted for age, sex, ethnicity, Townsend deprivation index, education, BMI, smoking status, alcohol consumption, physical activity, and dietary behaviors.

Abbreviations: FCRMD: first cardio-renal-metabolic disease; CRMM: cardio-renal-metabolic multimorbidity (the coexistence of two or three CRMDs); PhenoAge: phenotypic age; KDMAge: Klemera-Doubal method age; HR: hazard ratio; CI: confidence interval.

Table S6: Associations of the biological age acceleration with the trajectories of cardio-renal-metabolic multimorbidity of pattern A using the multi-state model, stratified by age groups

Indicator	< 65 years (*n* =234,476)			≥ 65 years (*n* = 44,451)			*P* for interaction
	Numbers of Cases	HR (95%CI)	*P* value	Numbers of cases	HR (95%CI)	*P* value	
**PhenoAge acceleration**							
Baseline to FCRMD	46,007	1.18 (1.17, 1.19)	< 0.001	18,086	1.17 (1.16, 1.19)	< 0.001	0.014
Baseline to death	4946	1.26 (1.23, 1.29)	< 0.001	2226	1.18 (1.14, 1.23)	< 0.001	0.005
FCRMD to CRMM	6028	1.21 (1.18, 1.23)	< 0.001	3484	1.29 (1.25, 1.32)	< 0.001	< 0.001
FCRMD to death	5277	1.14 (1.11, 1.16)	< 0.001	3424	1.11 (1.07, 1.14)	< 0.001	0.275
CRMM to death	1175	1.10 (1.05, 1.14)	< 0.001	1017	1.07 (1.02, 1.13)	0.006	0.530
**KDMAge acceleration**							
Baseline to FCRMD	46,007	1.21 (1.20, 1.22)	< 0.001	18,086	1.18 (1.16, 1.20)	< 0.001	< 0.001
Baseline to death	4946	1.17 (1.14, 1.21)	< 0.001	2226	1.11 (1.06, 1.16)	< 0.001	0.006
FCRMD to CRMM	6028	1.29 (1.26, 1.32)	< 0.001	3484	1.33 (1.29, 1.38)	< 0.001	0.047
FCRMD to death	5277	1.03 (1.00, 1.05)	0.057	3424	1.05 (1.01, 1.08)	0.007	0.641
CRMM to death	1175	1.02 (0.97, 1.07)	0.505	1017	1.05 (0.99, 1.11)	0.084	0.413

Estimates are presented per SD increase.

Models were adjusted for age, sex, ethnicity, Townsend deprivation index, education, BMI, smoking status, alcohol consumption, physical activity, and dietary behaviors.

Abbreviations: FCRMD: first cardio-renal-metabolic disease; CRMM: cardio-renal-metabolic multimorbidity (the coexistence of two or three CRMDs); PhenoAge: phenotypic age; KDMAge: Klemera-Doubal method age; HR: hazard ratio; CI: confidence interval.

Table S7: Associations of the biological age acceleration with the trajectories of cardio-renal-metabolic multimorbidity of pattern A using the multi-state model, stratified by sex

Indicator	Man (*n* = 122,796)			Female (*n* = 156,131)			*P* for interaction
	Numbers of Cases	HR (95%CI)	*P* value	Numbers of cases	HR (95%CI)	*P* value	
**PhenoAge acceleration**							
Baseline to FCRMD	34,011	1.19 (1.18, 1.20)	< 0.001	30,082	1.18 (1.17, 1.19)	< 0.001	0.264
Baseline to death	3467	1.28 (1.24, 1.31)	< 0.001	3705	1.21 (1.18, 1.25)	< 0.001	0.009
FCRMD to CRMM	5258	1.25 (1.23, 1.28)	< 0.001	4254	1.24 (1.21, 1.27)	< 0.001	0.058
FCRMD to death	5028	1.15 (1.12, 1.18)	< 0.001	3673	1.10 (1.07, 1.13)	< 0.001	0.017
CRMM to death	1308	1.08 (1.04, 1.13)	< 0.001	884	1.09 (1.05, 1.14)	< 0.001	0.827
**KDMAge acceleration**							
Baseline to FCRMD	34,011	1.21 (1.20, 1.22)	< 0.001	30,082	1.23 (1.22, 1.25)	< 0.001	0.006
Baseline to death	3467	1.19 (1.15, 1.23)	< 0.001	3705	1.13 (1.09, 1.18)	< 0.001	0.178
FCRMD to CRMM	5258	1.33 (1.30, 1.37)	< 0.001	4254	1.31 (1.27, 1.35)	< 0.001	0.031
FCRMD to death	5028	1.06 (1.03, 1.09)	< 0.001	3673	1.02 (0.98, 1.05)	0.356	0.118
CRMM to death	1308	1.05 (1.00, 1.10)	0.048	884	1.04 (0.97, 1.11)	0.316	0.713

Estimates are presented per SD increase.

Models were adjusted for age, sex, ethnicity, Townsend deprivation index, education, BMI, smoking status, alcohol consumption, physical activity, and dietary behaviors.

Abbreviations: FCRMD: first cardio-renal-metabolic disease; CRMM: cardio-renal-metabolic multimorbidity (the coexistence of two or three CRMDs); PhenoAge: phenotypic age; KDMAge: Klemera-Doubal method age; HR: hazard ratio; CI: confidence interval.

Table S8: Associations of biological age acceleration with the trajectories of cardio-renal-metabolic multimorbidity of pattern A using the multi-state model, stratified by Townsend deprivation index

Indicator	≤ Median (High economic status) (*n* = 139,197)			> Median (Low economic status) (*n* = 139,730)			*P* for interaction
	Numbers of Cases	HR (95%CI)	*P* value	Numbers of cases	HR (95%CI)	*P value*	
**PhenoAge acceleration**							
Baseline to FCRMD	31,027	1.18 (1.16, 1.19)	<0.001	33,066	1.19 (1.18, 1.20)	<0.001	0.020
Baseline to death	3501	1.23 (1.19, 1.27)	<0.001	3671	1.26 (1.23, 1.29)	<0.001	0.053
FCRMD to CRMM	4174	1.30 (1.27, 1.33)	<0.001	5338	1.20 (1.18, 1.23)	<0.001	<0.001
FCRMD to death	4013	1.11 (1.07, 1.14)	<0.001	4688	1.15 (1.12, 1.17)	<0.001	0.009
CRMM to death	941	1.04 (0.99, 1.10)	0.138	1251	1.11 (1.07, 1.16)	<0.001	0.027
**KDMAge acceleration**							
Baseline to FCRMD	31,027	1.19 (1.18, 1.21)	< 0.001	33,066	1.24 (1.22, 1.25)	<0.001	<0.001
Baseline to death	3501	1.13 (1.09, 1.17)	< 0.001	3671	1.19 (1.15, 1.23)	<0.001	0.020
FCRMD to CRMM	4174	1.37 (1.33, 1.41)	< 0.001	5338	1.29 (1.26, 1.33)	<0.001	0.001
FCRMD to death	4013	1.03 (1.00, 1.07)	0.043	4688	1.05 (1.02, 1.08)	<0.001	0.421
CRMM to death	941	1.02 (0.96, 1.09)	0.492	1251	1.06 (1.01, 1.12)	0.016	0.139

Estimates are presented per SD increase.

Models were adjusted for age, sex, ethnicity, Townsend deprivation index, education, BMI, smoking status, alcohol consumption, physical activity, and dietary behaviors.

Abbreviations: FCRMD: first cardio-renal-metabolic disease; CRMM: cardio-renal-metabolic multimorbidity (the coexistence of two or three CRMDs); PhenoAge: phenotypic age; KDMAge: Klemera-Doubal method age; HR: hazard ratio; CI: confidence interval.

Table S9: Associations of biological age acceleration with the trajectories of cardio-renal-metabolic multimorbidity of pattern A using the multi-state model, stratified by education level

Indicator	Low education level (*n* = 142,220)			High education level (*n* = 136,707)			*P* for interaction
	Numbers of Cases	HR (95%CI)	*P* value	Numbers of cases	HR (95%CI)	*P value*	
**PhenoAge acceleration**							
Baseline to FCRMD	36,486	1.19 (1.18, 1.20)	< 0.001	27,607	1.17 (1.15, 1.18)	< 0.001	< 0.001
Baseline to death	4038	1.25 (1.22, 1.28)	< 0.001	3134	1.24 (1.20, 1.28)	< 0.001	0.271
FCRMD to CRMM	6094	1.22 (1.20, 1.24)	< 0.001	3418	1.28 (1.25, 1.31)	< 0.001	0.002
FCRMD to death	5213	1.13 (1.10, 1.15)	< 0.001	3488	1.14 (1.10, 1.17)	< 0.001	0.636
CRMM to death	1457	1.09 (1.05, 1.13)	< 0.001	735	1.09 (1.03, 1.16)	0.002	0.993
**KDMAge acceleration**							
Baseline to FCRMD	36,486	1.22 (1.21, 1.23)	< 0.001	27,607	1.21 (1.19, 1.23)	< 0.001	0.033
Baseline to death	4038	1.17 (1.13 1.21)	< 0.001	3134	1.14 (1.10, 1.19)	< 0.001	0.878
FCRMD to CRMM	6094	1.32(1.29, 1.35)	< 0.001	3418	1.33 (1.29, 1.38)	< 0.001	0.313
FCRMD to death	5213	1.05 (1.02, 1.08)	< 0.001	3488	1.04 (1.00, 1.07)	0.035	0.994
CRMM to death	1457	1.06 (1.01 1.11)	0.010	735	1.01 (0.94, 1.08)	0.861	0.115

Estimates are presented per SD increase.

Models were adjusted for age, sex, ethnicity, Townsend deprivation index, education, BMI, smoking status, alcohol consumption, physical activity, and dietary behaviors.

Abbreviations: FCRMD: first cardio-renal-metabolic disease; CRMM: cardio-renal-metabolic multimorbidity (the coexistence of two or three CRMDs); PhenoAge: phenotypic age; KDMAge: Klemera-Doubal method age; HR: hazard ratio; CI: confidence interval.

Table S10: Associations of biological age acceleration with the trajectories of cardio-renal-metabolic multimorbidity of pattern A using the multi-state model, stratified by BMI

Indicator	Non-obese (BMI < 30 kg/m²) (*n* = 219,622)			Obese (BMI ≥ 30 kg/m²) (*n* = 59,305)			*P* for interaction
	Numbers of Cases	HR (95%CI)	*P* value	Numbers of cases	HR (95%CI)	*P* value	
**PhenoAge acceleration**							
Baseline to FCRMD	45,090	1.18 (1.17, 1.19)	<0.001	19,003	1.23 (1.22, 1.25)	< 0.001	<0.001
Baseline to death	5766	1.24 (1.21, 1.26)	<0.001	1406	1.28 (1.22, 1.34)	< 0.001	0.270
FCRMD to CRMM	5438	1.26 (1.24, 1.28)	<0.001	4074	1.25 (1.22, 1.28)	< 0.001	0.217
FCRMD to death	6450	1.13 (1.11, 1.16)	<0.001	2251	1.10 (1.06, 1.14)	< 0.001	0.046
CRMM to death	1275	1.08 (1.04, 1.13)	<0.001	917	1.10 (1.05, 1.15)	< 0.001	0.670
**KDMAge acceleration**							
Baseline to FCRMD	45,090	1.24 (1.23, 1.25)	<0.001	19,003	1.23 (1.21, 1.25)	<0.001	0.221
Baseline to death	5766	1.16 (1.13, 1.20)	<0.001	1406	1.08 (1.03, 1.15)	0.005	0.043
FCRMD to CRMM	5438	1.37 (1.33, 1.40)	<0.001	4074	1.31 (1.27, 1.35)	<0.001	0.002
FCRMD to death	6450	1.04 (1.01, 1.06)	0.002	2251	1.03 (0.99, 1.07)	0.199	0.151
CRMM to death	1275	1.01 (0.96, 1.06)	0.615	917	1.10 (1.03, 1.17)	0.004	0.047

Estimates are presented per SD increase.

Models were adjusted for age, sex, ethnicity, Townsend deprivation index, education, BMI, smoking status, alcohol consumption, physical activity, and dietary behaviors.

Abbreviations: FCRMD: first cardio-renal-metabolic disease; CRMM: cardio-renal-metabolic multimorbidity (the coexistence of two or three CRMDs); PhenoAge: phenotypic age; KDMAge: Klemera-Doubal method age; HR: hazard ratio; CI: confidence interval.

Table S11: Associations of biological age acceleration with the trajectories of cardio-renal-metabolic multimorbidity of pattern A using the multi-state model, stratified by smoking status

Indicator	Never-smoking (n = 158,072)			Ever-smoking (n = 120,855)			P for interaction
	Numbers of Cases	HR (95%CI)	P value	Numbers of cases	HR (95%CI)	P value	
**PhenoAge acceleration**							
Baseline to FCRMD	32,146	1.16 (1.15, 1.17)	< 0.001	31,947	1.22 (1.21, 1.24)	< 0.001	< 0.001
Baseline to death	3242	1.23 (1.19, 1.26)	< 0.001	3930	1.30 (1.27, 1.33)	< 0.001	0.002
FCRMD to CRMM	4307	1.24 (1.21, 1.27)	< 0.001	5205	1.26 (1.23, 1.28)	< 0.001	0.480
FCRMD to death	3694	1.10 (1.07, 1.13)	< 0.001	5007	1.18 (1.16, 1.21)	< 0.001	< 0.001
CRMM to death	862	1.14 (1.08, 1.20)	< 0.001	1330	1.08 (1.04, 1.12)	< 0.001	0.081
**KDMAge acceleration**							
Baseline to FCRMD	32,146	1.20 (1.19, 1.21)	< 0.001	31,947	1.25 (1.23, 1.26)	< 0.001	< 0.001
Baseline to death	3242	1.10 (1.06, 1.15)	< 0.001	3930	1.24 (1.20, 1.28)	< 0.001	< 0.001
FCRMD to CRMM	4307	1.31 (1.28, 1.35)	< 0.001	5205	1.35 (1.32, 1.39)	< 0.001	0.046
FCRMD to death	3694	0.98 (0.95, 1.02)	0.370	5007	1.11 (1.08, 1.14)	< 0.001	< 0.001
CRMM to death	862	1.01 (0.95, 1.08)	0.695	1330	1.07 (1.02, 1.12)	0.005	0.230

Estimates are presented per SD increase.

Models were adjusted for age, sex, ethnicity, Townsend deprivation index, education, BMI, smoking status, alcohol consumption, physical activity, and dietary behaviors.

Abbreviations: FCRMD: first cardio-renal-metabolic disease; CRMM: cardio-renal-metabolic multimorbidity (the coexistence of two or three CRMDs); PhenoAge: phenotypic age; KDMAge: Klemera-Doubal method age; HR: hazard ratio; CI: confidence interval.

Table S12: Associations of biological age acceleration with the trajectories of cardio-renal-metabolic multimorbidity of pattern A using the multi-state model, stratified by alcohol consumption

Indicator	Non-moderate alcohol consumption (n = 139,830)			Moderate alcohol consumption (n = 139,097)			P for interaction
	Numbers of Cases	HR (95%CI)	P value	Numbers of cases	HR (95%CI)	P value	
**PhenoAge acceleration**							
Baseline to FCRMD	33,768	1.20 (1.18, 1.21)	< 0.001	30,325	1.17 (1.16, 1.18)	< 0.001	<0.001
Baseline to death	3803	1.26 (1.22, 1.29)	< 0.001	3369	1.24 (1.20, 1.28)	< 0.001	0.273
FCRMD to CRMM	5312	1.23 (1.20, 1.25)	< 0.001	4200	1.27 (1.24, 1.30)	< 0.001	0.014
FCRMD to death	4788	1.13 (1.11, 1.16)	< 0.001	3913	1.13 (1.09, 1.16)	< 0.001	0.369
CRMM to death	1258	1.08 (1.04, 1.12)	< 0.001	934	1.11 (1.05, 1.16)	< 0.001	0.501
**KDMAge acceleration**							
Baseline to FCRMD	33,768	1.22 (1.21, 1.24)	< 0.001	30,325	1.21 (1.20, 1.22)	< 0.001	0.004
Baseline to death	3803	1.17 (1.13, 1.21)	< 0.001	3369	1.16 (1.12, 1.20)	< 0.001	0.614
FCRMD to CRMM	5312	1.33 (1.29, 1.36)	< 0.001	4200	1.33 (1.29, 1.37)	< 0.001	0.916
FCRMD to death	4788	1.06 (1.03, 1.09)	< 0.001	3913	1.03 (1.00, 1.06)	0.084	0.586
CRMM to death	1258	1.01 (0.96, 1.06)	0.763	934	1.09 (1.03, 1.16)	0.002	0.042

Estimates are presented per SD increase.

Models were adjusted for age, sex, ethnicity, Townsend deprivation index, education, BMI, smoking status, alcohol consumption, physical activity, and dietary behaviors.

Abbreviations: FCRMD: first cardio-renal-metabolic disease; CRMM: cardio-renal-metabolic multimorbidity (the coexistence of two or three CRMDs); PhenoAge: phenotypic age; KDMAge: Klemera-Doubal method age; HR: hazard ratio; CI: confidence interval.

Table S13: Associations of biological age acceleration with the trajectories of cardio-renal-metabolic multimorbidity of pattern A using the multi-state model, stratified by physical activity

Indicator	Low physical activity (≤3000 MET-min/week) (*n* = 163,528)			High physical activity (>3000 MET-min/week) (*n* = 115,399)			*P* for interaction
	Numbers of Cases	HR (95%CI)	*P* value	Numbers of cases	HR (95%CI)	*P value*	
**PhenoAge acceleration**							
Baseline to FCRMD	37,914	1.20 (1.18, 1.21)	< 0.001	26,179	1.16 (1.15, 1.18)	< 0.001	< 0.001
Baseline to death	4282	1.27 (1.24, 1.30)	< 0.001	2890	1.21 (1.17, 1.25)	< 0.001	0.041
FCRMD to CRMM	5969	1.24 (1.22, 1.27)	< 0.001	3543	1.24 (1.21, 1.27)	< 0.001	0.962
FCRMD to death	5189	1.13 (1.10, 1.15)	< 0.001	3512	1.14 (1.11, 1.17)	< 0.001	0.413
CRMM to death	1405	1.09 (1.05, 1.13)	< 0.001	787	1.10 (1.03, 1.17)	0.002	0.954
**KDMAge acceleration**							
Baseline to FCRMD	37,914	1.23 (1.22, 1.24)	< 0.001	26,179	1.20 (1.18, 1.21)	< 0.001	< 0.001
Baseline to death	4282	1.17 (1.13, 1.21)	< 0.001	2890	1.15 (1.11, 1.20)	< 0.001	0.976
FCRMD to CRMM	5969	1.33 (1.29, 1.36)	< 0.001	3543	1.32 (1.28, 1.37)	< 0.001	0.612
FCRMD to death	5189	1.04 (1.01, 1.07)	0.003	3512	1.05 (1.02, 1.09)	0.003	0.808
CRMM to death	1405	1.07 (1.02, 1.12)	0.008	787	1.01 (0.94, 1.08)	0.857	0.098

Estimates are presented per SD increase.

Models were adjusted for age, sex, ethnicity, Townsend deprivation index, education, BMI, smoking status, alcohol consumption, physical activity, and dietary behaviors.

Abbreviations: FCRMD: first cardio-renal-metabolic disease; CRMM: cardio-renal-metabolic multimorbidity (the coexistence of two or three CRMDs); PhenoAge: phenotypic age; KDMAge: Klemera-Doubal method age; MET: metabolic equivalent of task; HR: hazard ratio; CI: confidence interval.

Table S14: Associations of biological age acceleration with the trajectories of cardio-renal-metabolic multimorbidity of pattern A using the multi-state model, stratified by dietary behaviors

Indicator	Unhealthy dietary behaviors (*n* = 233.104)			Healthy dietary behaviors (*n* = 45,823)			*P* for interaction
	Numbers of Cases	HR (95%CI)	*P* value	Numbers of cases	HR (95%CI)	*P* value	
**PhenoAge acceleration**							
Baseline to FCRMD	53,368	1.19 (1.18, 1.20)	< 0.001	10,725	1.14 (1.12, 1.16)	< 0.001	< 0.001
Baseline to death	5906	1.26 (1.23, 1.29)	< 0.001	1266	1.18 (1.12, 1.24)	< 0.001	0.019
FCRMD to CRMM	8094	1.24 (1.22, 1.26)	< 0.001	1418	1.25 (1.20, 1.30)	< 0.001	0.743
FCRMD to death	7160	1.13 (1.11, 1.16)	< 0.001	1541	1.12 (1.07, 1.17)	< 0.001	0.502
CRMM to death	1867	1.09 (1.06, 1.13)	< 0.001	325	1.07 (0.98, 1.16)	0.156	0.537
**KDMAge acceleration**							
Baseline to FCRMD	53,368	1.23 (1.22, 1.24)	< 0.001	10,725	1.16 (1.14, 1.18)	< 0.001	< 0.001
Baseline to death	5906	1.17 (1.14, 1.20)	< 0.001	1266	1.13 (1.06, 1.20)	< 0.001	0.970
FCRMD to CRMM	8094	1.32 (1.30, 1.35)	< 0.001	1418	1.34 (1.28, 1.42)	< 0.001	0.275
FCRMD to death	7160	1.05 (1.03, 1.07)	< 0.001	1541	1.02 (0.97, 1.08)	0.423	0.607
CRMM to death	1867	1.05 (1.01, 1.10)	0.018	325	1.02 (0.92, 1.13)	0.655	0.301

Estimates are presented per SD increase.

Models were adjusted for age, sex, ethnicity, Townsend deprivation index, education, BMI, smoking status, alcohol consumption, physical activity, and dietary behaviors.

Abbreviations: FCRMD: first cardio-renal-metabolic disease; CRMM: cardio-renal-metabolic multimorbidity (the coexistence of two or three CRMDs); PhenoAge: phenotypic age; KDMAge: Klemera-Doubal method age; HR: hazard ratio; CI: confidence interval.

Table S15: Sensitivity analysis of associations between biological age acceleration and trajectories of cardio-renal-metabolic multimorbidity

	HRs (95%CIs)					
	**Baseline to FCRMD**	**Baseline to death**	**FCRMD to CRMM**	**FCRMD to death**	**CRMM to death**	**Baseline to CRMM**
**PhenoAge acceleration**						
Additional time intervals[a]						
0.5 years	**1.18 (1.17, 1.19)**	**1.25 (1.23, 1.27)**	**1.24 (1.22, 1.26)**	**1.13 (1.11, 1.15)**	**1.09 (1.06, 1.12)**	
1 year	**1.18 (1.17, 1.19)**	**1.25 (1.23, 1.27)**	**1.24 (1.22, 1.26)**	**1.13 (1.11, 1.15)**	**1.09 (1.06, 1.12)**	
3 years	**1.18 (1.17, 1.19)**	**1.25 (1.23, 1.27)**	**1.23 (1.22, 1.25)**	**1.13 (1.11, 1.15)**	**1.09 (1.06, 1.13)**	
5 years	**1.18 (1.17, 1.19)**	**1.26 (1.24, 1.28)**	**1.23 (1.21, 1.25)**	**1.13 (1.11, 1.16)**	**1.08 (1.05, 1.12)**	
Adding a transition directly from baseline to CRMM	**1.18 (1.17, 1.18)**	**1.25 (1.22, 1.27)**	**1.25 (1.23, 1.27)**	**1.13 (1.11, 1.15)**	**1.09 (1.06, 1.12)**	**1.36 (1.32, 1.41)**
Including only White participants	**1.19 (1.18, 1.19)**	**1.25 (1.22, 1.27)**	**1.25 (1.23, 1.27)**	**1.13 (1.11, 1.15)**	**1.09 (1.05, 1.12)**	
Excluding participants diagnosed with any CRMD within two years after enrollment	**1.17 (1.16, 1.17)**	**1.20 (1.17, 1.22)**	**1.23 (1.21, 1.25)**	**1.12 (1.10, 1.14)**	**1.09 (1.05, 1.13)**	
Further adjusting for the use of cholesterol-lowering and antihypertensive medications	**1.18 (1.17, 1.19)**	**1.25 (1.22, 1.27)**	**1.24 (1.22, 1.26)**	**1.13 (1.11, 1.15)**	**1.09 (1.06, 1.12)**	
Excluding participants with abnormal baseline glucose, glycated hemoglobin, eGFR, or albuminuria	**1.14 (1.14, 1.15)**	**1.24 (1.21, 1.26)**	**1.19 (1.16, 1.21)**	**1.16 (1.13, 1.18)**	**1.11 (1.07, 1.16)**	
**KDMAge acceleration**						
Different intervals						
0.5 years	**1.22 (1.21, 1.23)**	**1.17 (1.14, 1.19)**	**1.33 (1.30, 1.35)**	**1.05 (1.02, 1.07)**	**1.04 (1.00, 1.08)**	
1 year	**1.22 (1.21, 1.23)**	**1.17 (1.14, 1.20)**	**1.32 (1.30, 1.35)**	**1.05 (1.02, 1.07)**	**1.04 (1.00, 1.08)**	
3 years	**1.22 (1.21, 1.23)**	**1.17 (1.14, 1.20)**	**1.32 (1.29, 1.35)**	**1.05 (1.03, 1.07)**	**1.04 (1.00, 1.09)**	
5 years	**1.21 (1.20, 1.22)**	**1.18 (1.15, 1.21)**	**1.32 (1.29, 1.34)**	**1.05 (1.03, 1.07)**	**1.04 (1.00, 1.09)**	
Adding a transition directly from baseline to CRMM	**1.21 (1.20, 1.22)**	**1.16 (1.13, 1.19)**	**1.34 (1.31, 1.37)**	**1.05 (1.02, 1.07)**	**1.04 (1.00, 1.08)**	**1.58 (1.51, 1.65)**
Including only White participants	**1.21 (1.20, 1.22)**	**1.17(1.14. 1.20)**	**1.33 (1.30. 1.36)**	**1.05 (1.03. 1.07)**	**1.05 (1.00. 1.09)**	

Excluding participants diagnosed with any CRMD within two years after enrollment	**1.20 (1.19, 1.21)**	**1.13 (1.10, 1.16)**	**1.32 (1.29, 1.35)**	**1.04 (1.02, 1.06)**	1.03 (0.99, 1.08)
Further adjusting for the use of cholesterol-lowering and antihypertensive medications	**1.22 (1.21, 1.23)**	**1.16 (1.13, 1.19)**	**1.33 (1.30, 1.35)**	**1.05 (1.02, 1.07)**	**1.04 (1.00, 1.08)**
Excluding participants with abnormal baseline glucose, glycated hemoglobin, eGFR, or albuminuria	**1.17 (1.16, 1.18)**	**1.15 (1.12, 1.18)**	**1.26 (1.23, 1.29)**	**1.06 (1.04, 1.09)**	1.03 (0.98, 1.09)

Estimates are presented per SD increase.

Models were adjusted for age, sex, ethnicity, Townsend deprivation index, education, BMI, smoking status, alcohol consumption, physical activity, and dietary behaviors.

[a]For participants entering different states on the same date, the entry date for the prior state was calculated using four additional time intervals: 0.5, 1, 3, and 5 years.

Abbreviations: FCRMD: first cardio-renal-metabolic disease; CRMM: cardio-renal-metabolic multimorbidity (the coexistence of two or three CRMDs); PhenoAge: phenotypic age; KDMAge: Klemera-Doubal method age; HR: hazard ratio; CI: confidence interval.
